# Season of birth is associated with increased risk of atopic dermatitis in Japanese infants: a retrospective cohort study

**DOI:** 10.1186/s13223-020-00443-z

**Published:** 2020-05-29

**Authors:** Yu Kuwabara, Ritsue Nii, Keiko Tanaka, Eiichi Ishii, Mizuho Nagao, Takao Fujisawa

**Affiliations:** 1 Department of Pediatrics, Ehime University Graduate School of Medicine, Shitsukawa, Toon, Ehime 791-0295 Japan; 2 Allergy Center and Institute for Clinical Research, National Hospital Organization Mie National Hospital, Osato-kubota, Tsu, Mie 514-0125 Japan; 3 Department of Pediatrics, Shiroko Clinic, Minami-ejima, Suzuka, Mie 510-0235 Japan; 4 Department of Epidemiology and Preventive Medicine, Ehime University Graduate School of Medicine, Shitsukawa, Toon, Ehime 791-0295 Japan

**Keywords:** Atopic dermatitis, Food allergy, Season of birth

## Abstract

**Background:**

Several epidemiological studies have examined the possibility of a relationship between season of birth and atopic dermatitis (AD) and food allergy (FA), yet their results are contradictory. We investigated the association between season of birth and risk of AD and FA in Japanese infants.

**Methods:**

Study subjects were 612 newborn infants born at a single obstetric/pediatric clinic without perinatal diseases. Season of birth was classified as spring (March–May), summer (June–August), autumn (September–November) or winter (December–February). AD was diagnosed according to the United Kingdom Working Party’s criteria. FA was defined as present if there was a history of immediate allergic symptoms within 2 h after ingestion of a food. Specific IgE to the corresponding food was also assessed to support the diagnosis. We assessed the association between season of birth and risk of AD and FA using Cox proportional hazard models.

**Results:**

We identified a total of 365 cases of AD occurring during 3659 person-months of follow-up. Compared with summer birth, autumn, winter, and spring birth were significantly positively associated with the risk of AD: adjusted HRs (95% CIs) were 2.67 (1.96–3.63), 1.42 (1.03–1.95), and 1.43 (1.04–1.98), respectively. We identified a total of 23 cases of physician-diagnosed FA occurring during 6815 person-months of follow-up.

**Conclusions:**

Being born in the summer is associated with a lower risk of AD compared to other seasons of birth. The low incidence of FA in our cohort group made it difficult to establish a valid association between FA and season of birth as the statistical power was low.

## Background

The prevalence of atopic dermatitis (AD) continues to increase worldwide; it is already the most common chronic inflammatory skin disease in childhood [1, 2]. Genetic and environmental factors such as birthweight, socioeconomic status, gestational age, multiparity, and a family history of allergic disorders have been reported to be associated with the development and prevalence of AD [3–5].

Previous studies have examined the association between season of birth (SoB) and AD in infants, children, and adolescents [5–13]. In a Japanese cohort study in infants, birth in spring compared with birth in any other season was inversely associated with the risk of AD, whereas birth in autumn or winter compared with birth in any other season was associated with an increased risk of AD [7]. A Korean cross-sectional study showed that, compared with birth in summer (April to September), birth in winter (October to March) was positively associated with the prevalence of ever having experienced AD symptoms in children aged 8 years [9]. In a birth cohort study in Sweden, atopic disease was more common among children who were born in the autumn or winter than among those born in spring or summer [12].

Recently it has been proposed that severe AD in infancy might be a risk factor for the subsequent development of food allergy (FA), because food allergens may penetrate via antigen-presenting cells in eczematous skin [14–16]. Several studies have examined the association between season of birth and the prevalence of food allergy [17–21]. In Australia, autumn/winter births were associated with an increased prevalence of food allergy compared to spring/summer births in children aged 0 to 4 years [19]. In contrast, a cross-sectional study in Japan showed no association between SoB and the prevalence of FA in infants [21]. In a study using data from the National Health and Nutrition Examination Survey III in the U.S., a positive association was observed between autumn birth and the prevalence of FA only in children with eczema, but not in those without eczema [20].

Not only children with AD and FA but also their family members have a lower quality of life due to food and social restrictions related to avoidance of allergens and financial costs of care [22, 23]. Clarifying the role of SoB in the development of AD and FA will be relevant to efforts to monitor and treat these allergic diseases from their earliest stages. In the present birth cohort study, we examine the association between birth season and the risk of AD and FA in Japanese infants.

## Methods

### Study population

This study was a retrospective, single-center, hospital-based birth cohort study. We collected information on all babies who were born in an obstetric/pediatric clinic located in a rural area in Mie Prefecture on the main island of Japan, who were born without perinatal diseases between 1 September 2013 and 31 August 2014 and whose mothers visited the clinic for a routine health checkup 1 month after birth. At this clinic, infants receive health check-ups or vaccinations at 1, 2, 3, 4, 5, 7, 10, and 12 months after birth. At each of these visits they were also checked for eczema and FA.

### Measurements

We obtained data on gestational age, birth weight and length, birth month, sex, mode of delivery, number of older siblings, presence of eczema, extent of eczema, AD, use of emollients, breast feeding (exclusive breast feeding or mixed feeding or exclusive cow’s milk formula feeding), intake of complementary food (rice, vegetables, soy, fish, fruit, wheat, milk and hen’s eggs), food elimination, food sensitization, food-induced symptoms and FA from medical records. Each infant’s SoB was classified as spring (March–May), summer (June–August), autumn (September–November) or winter (December–February). We defined AD according to the United Kingdom Working Party’s criteria [24]. FA was defined as present if there was a history of immediate allergic symptoms such as urticaria, erythema or itching of the skin, eyes, nose or mouth, vomiting, cough, wheeze and hypotension within 2 h after ingestion of a food. Specific IgE to the corresponding food was also assessed to support the diagnosis. Delayed allergic symptoms (> 2 h between ingestion and onset) may indicate non-IgE-mediated food allergy and were not included as FA since IgE-mediated and non-IgE-mediated food allergies have different pathogenesis. Diagnosis of AD and FA was made by a single experienced pediatrician.

### Statistical analysis

Person-months were counted from birth until the age in months at the first diagnosis with an outcome under study (AD or FA) or at the end of the follow-up period (maximum 12 months of age), whichever came first. We used Cox proportional hazard models to assess the association between SoB and AD or FA in children. In the analysis for AD, the multivariate hazard ratios (HRs) were adjusted for sex (male or female), birthweight (continuous, g), older siblings (0 or ≥ 1), cesarean section (yes or no), and use of emollients at 1 month of age (yes or no). In the analysis for FA, we adjusted for sex (male or female), birthweight (continuous, g), older siblings (0 or ≥ 1), atopic dermatitis at 1 month (yes or no), and use of emollients at 1 month of age (yes or no). All statistical analyses were performed using the SAS software package version 9.4 (SAS Institute, Inc., Cary, NC, USA). Two-sided p-values < 0.05 were considered statistically significant. The study protocol was approved by the Ethics Committees of Mie National Hospital.

## Results

We identified a total of 365 cases of AD occurring during 3659 person-months of follow-up (mean 6 months; median 4 months). We also identified a total of 23 cases of physician-diagnosed FA occurring during 6815 person-months of follow-up (mean 11 months; median 12 months), including 11 hen’s egg, 8 milk, 1 wheat, 2 soybean and 2 fish allergies. One infant had both a soybean allergy and a fish allergy. Of the 23 children with FA, 19 had positive specific IgE. Although two cases had negative specific IgE and the test was not available in one case of fish allergy and not performed in one case of hen’s egg allergy, all 23 subjects exhibited immediate allergic symptoms after sole ingestion of the food. The characteristics of the study population are provided in Table 1. Mean birthweight was 3067.0 g, and about 53% of children had no siblings at birth. Approximately 10% of the children were born by cesarean section.

**Table 1:** Distribution of selected characteristics in 612 children

Variable	No. (%) or mean ± SD
Male sex	269 (44.0)
Birthweight, g, mean ± SD	3067.0 ± 394.1
Older siblings	
0	323 (52.8)
≥ 1	289 (47.2)
Cesarean birth	58 (9.5)
Use of emollient at 1 month of age	76 (12.4)
Atopic dermatitis at 1 month of age	106 (17.3)
Season of birth	
Spring	158 (25.8)
Summer	143 (23.4)
Autumn	159 (26.0)
Winter	152 (24.8)

*SD* standard deviation

Crude and adjusted HRs and 95% confidence intervals (CIs) for AD or FA in children in relation to SoB are shown in Table 2. Compared with summer birth, autumn, winter, and spring birth were significantly positively associated with the risk of AD: adjusted HRs (95% CIs) were 2.67 (1.96–3.63), 1.42 (1.03–1.95), and 1.43 (1.04–1.98), respectively. No association was observed between birth season and the risk of FA, however.

**Table 2:** Adjusted hazard ratios (HRs) and 95% confidence intervals (CIs) for atopic dermatitis and food allergy according to season of birth

	Atopic dermatitis				Food allergy			
	No. of events	Person-months of follow-up	Crude HR (95% CI)	Adjusted HR^a^ (95% CI)	No. of events	Person-months of follow-up	Crude HR (95% CI)	Adjusted HR^b^ (95% CI)
Spring	86	1056	1.29 (0.94–1.78)	1.43 (1.04–1.98)	7	1808	1.05 (0.35–3.12)	0.95 (0.32–2.85)
Summer	68	1077	1.00	1.00	6	1632	1.00	1.00
Autumn	117	671	2.18 (1.61–2.95)	2.67 (1.96–3.63)	6	1663	1.02 (0.33–3.16)	0.98 (0.32–3.07)
Winter	94	855	1.60 (1.17–2.19)	1.42 (1.03–1.95)	4	1712	0.64 (0.18–2.25)	0.58 (0.16–2.10)

*HR* hazard ratio, *CI* confidence interval

^a^Adjustment for sex, birthweight, siblings, cesarean birth, and use of emollient at 1 month of age

^b^Adjustment for sex, birthweight, siblings, atopic dermatitis at 1 month, and use of emollient at 1 month of age

## Discussion

The present study found that, compared with summer birth, autumn, winter, and spring birth were significantly positively associated with the risk of AD. On the other hand, there was no association between SoB and risk of FA.

A cross-sectional study in 14,669 Japanese schoolchildren aged 7–15 years showed that being born in autumn or winter was positively associated with the prevalence of eczema before 6 months of age [11]. Another web-based cross-sectional study also demonstrated that, compared with summer birth, autumn or winter birth was associated with an increased prevalence of current eczema in 23,662 Japanese children aged 6–12 years [8]. Our findings are in agreement with these findings. Previous studies, however, on the association between SoB and AD are not directly comparable to our study because of differences in the study designs, study populations, diagnostic criteria for AD, age of the study subjects, and controlling for confounding factors. For example, some of these studies accepted self-reported symptoms as indicative of AD [11] while others accepted positive answers to the International Study of Asthma and Allergies in Childhood questions [8–10] and still others required a physician diagnosis [6].

The underlying mechanisms of the association between SoB and AD have not yet been clarified. DNA methylation has been reported as one possible mechanism, given that DNA methylation patterns in the whole blood suggest that seasonal exposures might cause epigenetic changes in immune responses [25]. As newborn infants, children born in spring, autumn and winter have less sunlight exposure than those born in summer, resulting in lower vitamin D levels in early life. Ultraviolet B in particular may decrease inflammation, enforce skin barrier function, and reduce the risk of AD [13]. Vitamin D has pleiotropic effects on immune cells such as regulatory T cells, mast cells, and B cells, and can suppress allergic inflammation by producing IL-10 and suppressing allergen-specific IgE production [16, 26]. Alternatively, the seasonality of viral and other environmental exposures might influence immune development [27]. For AD, exposure to cold and dry weather might also contribute to the development of AD [5, 13, 28]. Horimukai et al. reported that daily application of moisturizer during the first 32 weeks of life reduced the risk of AD in infants [29].

Our study has several strengths. First, we used data from medical records retrospectively, which prevented recall bias. Second, our outcome definition for FA was based on pediatrician diagnosis, whereas that for AD was based on the UKWP diagnostic criteria. This prevented overestimation of FA and AD which can occur with parent-reported data [30, 31].

Our study has some potential limitations. Because the present study was limited to one clinic in a rural area of Japan, our findings are not applicable to the general population. In addition, the observation period was relatively short. Our definition of FA was based on physician diagnosis in response to a history of immediate objective allergic symptoms after ingestion of a food. We did not perform oral food challenges on children whose parents had eliminated suspected foods from their diets. It is possible that misclassification of outcome occurred. However, such misclassification of outcome is likely to be non-differential, that is, to be similar across exposure categories, and would tend to reduce the observed associations. Further, we were not able to confirm diagnosis of FA in four subjects with negative sensitization to their suspected foods as their parents did not consent to an oral food challenge because of the risk of anaphylaxis. In a sensitivity analysis excluding these four subjects, however, the results were similar to those in the original analysis. In the present study, the number of subjects who developed FA was small, leading to low statistical power. Statistical power calculation revealed that a sample size of 7104 subjects (370 events) would allow us to detect an association between SoB and the risk of FA for a HR 0.64 with a power of 80%. Because the low statistical power of the present analysis prevents a statistically valid conclusion, we could not suggest a valid association between FA and SoB in this study. Finally, although we did control for several potential confounding factors, our results remain confounded by unknown or residual factors.

## Conclusions

To our knowledge, this is the first report to examine the association between FA defined according to a physician’s diagnosis and SoB in Japanese infants. We found that, compared with summer birth, spring, autumn, or winter birth increased the risk of AD in Japanese infants, while the low incidence of FA in our cohort group made it difficult to establish a valid association between FA and season of birth as the statistical power was low. Further epidemiological research is needed to accumulate evidence on the association between SoB and AD and FA in addition to clarifying the mechanisms underlying these relationships.

## Data Availability

The datasets used and analyzed during this study are available from the corresponding author on reasonable request.

## Abbreviations

AD: Atopic dermatitis
CI: Confidence interval
FA: Food allergy
HR: Hazard ratio
SoB: Season of birth
